# A Green, Expeditious, One-Pot Synthesis of 3, 4-Dihydropyrimidin-2(1H)-ones Using a Mixture of Phosphorus Pentoxide-Methanesulfonic Acid at Ambient Temperature

**DOI:** 10.5402/2012/415645

**Published:** 2012-08-08

**Authors:** Amulrao Borse, Mahesh Patil, Nilesh Patil, Rohan Shinde

**Affiliations:** ^1^School of Chemical Sciences, North Maharashtra University, Jalgaon 425 001, India; ^2^Department of Pharmacology, R. C. Patel Institute of Pharmaceutical Education & Research, Shirpur 425 405, India

## Abstract

An expeditious, one-pot method for the synthesis of 3,4-dihydropyrimidin-2(1H)-ones using a mixture of phosphorus pentoxide-methanesulfonic acid (Eaton's reagent) at room temperature under solvent-free conditions is described. The salient features of this method include short reaction time, green aspects, high yields, and simple procedure.

## 1. Introduction

The widespread interest in 3,4-dihydropyrimidin-2(1H)-ones, Biginelli compounds, has resulted in enormous efforts towards the synthesis of this biologically important moiety. Several methods have been developed for the synthesis of these compounds, but most of these protocols involve expensive reagents, strong acid catalysts, solvents, of prolonged reaction time and even then provide the products in unsatisfactory yields. With the current global awareness of developing environmentally friendly technologies, it is a need to perform a reaction in neat and nonhazardous conditions for providing a green approach towards organic synthesis [1]. Therefore, it was decided to develop an efficient method for the synthesis of Biginelli compounds. In this communication, we report a straightforward and simple procedures for the synthesis of 3,4-dihydropyrimidin-2(1H)-ones using a mixture of phosphorus pentoxide-methanesulfonic acid (Eaton's reagent). 

Eaton's reagent (1 : 10 phosphorus pentoxide in methanesulfonic acid) is an inexpensive and commercially available substance synthesized by Eaton in 1973 and found to be a good alternative to polyphosphoric acid which enables the drawbacks of many traditional catalysts to be overcome, because it has a much lower viscosity, it is easier to handle, and no complex separation procedures need to be employed [2]. Many processes that employ a mixture of P_2_O_5_/MeSO_3_H are not only more economical, but also they are more environmentally friendly and offer a number of distinct advantages such as safe in industrial scale, no additional solvent required, chlorine-free, rapid reactions, and high-purity products with excellent yields. The distinctive physical and chemical properties of Eaton's reagent make it a very useful substance in many different reactions with different applications. The mixture of P_2_O_5_/MeSO_3_H is particularly effective for ring closures. McGarry and Detty successfully used this reagent in cycloacylation reactions for producing chromones and flavones [3]; recently, Zewge and coworkers used Eaton's reagent to promote the cyclization of aniline derivatives to produce 4-quinolones [4]. P_2_O_5_/MeSO_3_H offers a simple means of producing poly(benzimidazoles) from o-phenylenediamines and aromatic carboxylic acids [5]. Kaboudin and Abedi employed this system for synthesis of aryl mesylates [6].

**Scheme 1:**
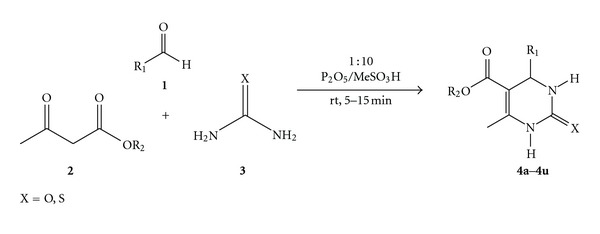
One-pot synthesis of 3,4-dihydropyrimidin-2(1H)-ones using Eaton's reagent.

In continuation of our efforts on developing environmentally benign, green methodologies for biologically active organic compounds [7–9], herein we report a rapid, ambient temperature, and solvent-free method for the synthesis of 3,4-dihydropyrimidin-2(1H)-ones using Eaton's reagent. 3,4-dihydropyrimidin-2(1H)-ones, Biginelli compounds, have been synthesized by condensing aldehyde, ethylacetoacetate and urea or thiourea under acidic conditions [10]. Methanesulfonic acid [11] and phosphorus pentoxide [12] have been used as catalysts in the past decade. Both of these transformations require conventional heating and use of organic solvents. In case of methanesulfonic acid, the reaction mixture is refluxed for 6-7 h using ethanol as solvent, and in phosphorus pentoxide, it is refluxed for 3-4 h. In view of these results, it was decided to use Eaton's reagent (P_2_O_5_ and CH_3_SO_3_H) under solvent-free conditions at room temperature for the synthesis of 3,4-dihydropyrimidin-2(1H)-ones. (Scheme 1). 

The synthesis of functionalized 3,4-dihydropyrimidin-2(1H)-one derivatives is the area of interest because a large number of biologically active molecules contain this moiety. Many dihydropyrimidinones and their derivatives are pharmacologically important as they possess antitumor, antibacterial, and antiviral properties; they have also emerged as integral backbones of several calcium-channel blockers, vasorelaxants, antihypertensive, and antimitotic agents [13–17]. The literature survey reveals that numerous methods have been developed for the synthesis of 3,4-dihydropyrimidin-2(1H)-ones by three-component cyclocondensation of aldehyde, urea, and ethylacetoacetate, which comprises the use of ionic liquids [18], microwave irradiation [19], ultrasound irradiation [20], BF_3_·OEt_2_ [21], NiCl_2_·6H_2_O and FeCl_3_·6H_2_O [22], CoCl_2_·6H_2_O [23], BiCl_3_ [24], InCl_3_ [25], and InBr_3_ [26]. Zn(OTf)_2_ [27], Cu(OTf)_2_ [28], Bi(OTf)_3_ [29], p-TSA [30], silica sulphuric acid [31], potassium hydrogen sulphate [32], formic acid [33], chloroacetic acid [34], chlorosulfonic acid [35], P_2_O_5_/SiO_2_ [36], TFA [37], CF_3_COONH_4_ [38], p-TSA in biphasic media [39], ZnCl_2_ [40], and I_2_ [41].

## 2. Result and Discussion

In our recent study about the synthesis of Bis(indolyl)methanes under mild conditions, we found that reagent works extremely well for the coupling reaction. In this paper, herein we employed the reagent in a multicomponent, one-pot reaction for the synthesis of 3,4-dihydropyrimidin-2(1H)-ones at room temperature. Eaton's reagent is a colorless, odorless liquid mixture of nonoxidizing methanesulfonic acid and a powerful dehydrating agent phosphorus pentoxide. The addition of phosphorus pentoxide increases the solubility of organic compounds in methanesulfonic acid; this was introduced by Eaton and has been used enormously in organic synthesis. 

In order to standardize the reaction conditions for the condensation reaction, it was decided to synthesize 3,4-dihydropyrimidin-2(1H)-one (**4a)** from benzaldehyde **(1a)**, urea, and ethylacetoacetate using a mixture of P_2_O_5_/MeSO_3_H, and we found that the reaction is fast when compared to other reported methods. The results are compared with the reported methods, and it is clear from Table 1 that the present method is more efficient. 

To optimize the reaction condition, the condensation reaction was performed under different conditions (Table 2). In the presence of Eaton's reagent (2 mmol for each operation), initially 4-chlorobenzaldehyde **(1k)** was used as model for the reaction with ethylacetoacetate and urea. The reaction carried out at room temperature using ethanol as solvent required 2.3 h for completion, and the product **(4k)** was obtained in 60% yield (Entry 1). When the reaction was performed by using ethanol under refluxing conditions, it required 2 h and provided the product in 65% yield (Entry 2). When the reaction was performed without any solvent at room temperature using 2 mmol of Eaton's reagent, the reaction was completed in 5 min, and the product **(4k)** was obtained in 85% yield (Entry 3).

To explore the scope and limitations of this reaction, we extended the procedure to various aromatic aldehydes carrying either electron-releasing or electron-withdrawing substituents in the ortho-, meta-, and para-positions. We have also synthesized the compounds with thiourea and methylacetoacetate, and we found that the reaction proceeds very efficiently with all the cases, and the products are obtained in high yields (Table 3).

## 3. Experimental

Eaton's reagent (7.7/92.3% by weight of P_2_O_5_/MeSO_3_H) was purchased from Sigma-Aldrich. All melting points were recorded in open capillaries. The purity of the compounds was checked by TLC on silica gel G (Merck). ^1^H NMR spectra were recorded on Varian 300 MHz instrument, in DMSO-*d*_6_ using TMS as the internal standard. IR spectra were obtained using a Nujol for solids on a Perkin-Elmer-1710 spectrophotometer. Mass spectra were recorded on Thermo Finnigan (Model-LCQ Advantage MAX) mass spectrometer.

### 3.1. General Method for the Synthesis of 3,4-Dihydropyrimidin-2(1H)-ones **(4a–4u)**

A mixture of aromatic aldehyde **1** (1 mmol), ethylacetoacetate **2** (1 mmol), and urea **3 **(1.5 mmol) was stirred with Eaton's reagent (2 mmol) at room temperature for appropriate time (mentioned in Table 3). After completion of the reaction (monitored by TLC), the reaction mass was transferred to an excess saturated sodium carbonate solution. The solid products separated out, were filtered, and washed with sufficient water and dried. The crude products on recrystallization from ethanol provided 3,4-dihydropyrimidin-2(1H)-ones **(4a–4u)** in 75–96% yield.

### 3.2. Spectral Data for the Selected Compounds

#### 3.2.1. ****5-Ethoxycarbonyl-4-(phenyl)-6-methyl-3,4-dihydropyrimidin-2(1H)-one (4a) 

Mp 202–204°C; IR(nujol) cm^−1^: 3244 (NH), 3108 (NH), 1729 (C=O), 1645 (C=C), 1460 (CH); ^1^H NMR (300 MHz, DMSO-*d*_6_): *δ*_H_ 9.18 (br s, 1H, NH), 7.73 (br s, 1H, NH), 7.21–7.34 (m, 5H, ArH), 5.13 (d, 1H, *J* = 3.3 Hz, CH), 3.97 (q, 2H, *J* = 7.15 Hz, OCH_2_), 2.24 (s, 3H, CH_3_), 1.08 (t, 3H, *J* = 7.15 Hz, CH_3_); MS (*m*/*z*): 260 (M^+^). 

#### 3.2.2. ****5-Ethoxycarbonyl-4-(2,3-dichlorophenyl)-6-methyl-3,4-dihydropyrimidin-2(1H)-one (4d)

Mp 248–250°C; IR(nujol) cm^−1^: 3357 (NH), 3108 (NH), 1696 (C=O), 1646 (C=C), 1459 (CH); ^1^H NMR (300 MHz, DMSO-*d*_6_): *δ*_H_ 9.32 (br s, 1H, NH), 7.78 (br s, 1H, NH), 7.27–7.56 (m, 3H, ArH), 5.67 (d, 1H, *J* = 2.4 Hz, CH), 3.88 (q, 2H, *J* = 6.9 Hz, OCH_2_), 2.29 (s, 3H, CH_3_), 0.96 (t, 3H, *J* = 7.15 Hz, CH_3_); MS (*m*/*z*): 329 (M^+^). 

#### 3.2.3. ****5-Ethoxycarbonyl-4-(2,4-dichlorophenyl)-6-methyl-3,4-dihydropyrimidin-2(1H)-one (4 g)

Mp 252–254°C; IR(nujol) cm^−1^: 3359 (NH), 3108 (NH), 1716 (C=O), 1640 (C=C), 1459 (CH); ^1^H NMR (300 MHz, DMSO-*d*_6_): *δ*_H_ 9.31 (br s, 1H, NH), 7.76 (br s, 1H, NH), 7.29–7.56 (m, 3H, ArH), 5.58 (s, 1H,CH), 3.88 (q, 2H, *J* = 7.15Hz, OCH_2_), 2.28 (s, 3H, CH_3_), 0.99 (t, 3H, *J* = 6.9Hz, CH_3_); MS (*m*/*z*): 329 (M^+^). 

#### 3.2.4. ****5-Ethoxycarbonyl-4-(4-bromophenyl)-6-methyl-3,4-dihydropyrimidin-2(1H)-one (4 h)

Mp 216–218°C; IR(nujol) cm^−1^: 3345 (NH), 3110 (NH), 1704 (C=O), 1645 (C=C), 1462 (CH); ^1^H NMR (300 MHz, DMSO-*d*_6_): *δ*_H_ 9.20 (br s, 1H, NH), 7.73 (br s, 1H, NH), 7.48 (d, 2H, *J* = 8.1 Hz, ArH), 7.14 (d, 2H, *J* = 8.1 Hz, ArH), 5.07 (d, 1H, *J* = 2.8 Hz, CH), 3.93 (q, 2H, *J* = 6.9 Hz, OCH_2_), 2.20 (s, 3H, CH_3_), 1.04 (t, 3H, *J* = 7.15 Hz, CH_3_); MS (*m*/*z*): 339 (M^+^). 

#### 3.2.5. ****5-Ethoxycarbonyl-4-(4-hydroxyphenyl)-6-methyl-3,4-dihydropyrimidin-2(1H)-one (4i) 

Mp 226–228°C; IR(nujol) cm^−1^: 3507 (NH), 3108 (NH), 1682 (C=O), 1645 (C=C), 1460 (CH); ^1^H NMR (300 MHz, DMSO-*d*_6_): *δ*_H_ 9.06 (br s, 1H, NH), 7.57 (br s, 1H, NH), 7.02 (d, 2H, ArH), 6.67 (d, 2H, ArH), 5.03 (d, 1H,*J* = 2.9 Hz, CH), 3.97 (q, 2H, *J* = 7.15 Hz, OCH_2_), 2.22 (s, 3H, CH_3_), 1.09 (t, 3H, *J* = 7.15 Hz, CH_3_); MS (*m*/*z*): 277 (M^+^+H). 

#### 3.2.6. ****5-Mthoxycarbonyl-4-(4-fluorophenyl)-6-methyl-3,4-dihydropyrimidin-2(1H)-one (4n)

Mp 208–210°C; IR(nujol) cm^−1^: 3326(NH), 3204 (NH), 1695 (C=O), 1666 (C=C), 1460 (CH); ^1^H NMR (300 MHz, DMSO-*d*_6_): *δ*_H_ 9.24 (br s, 1H, NH), 7.76 (br s, 1H, NH), 7.11–7.27 (m, 4H, ArH), 5.13 (d, 1H,*J* = 2.9 Hz, CH), 3.51 (s, 3H, CH_3_O), 2.24 (s, 3H, CH_3_); MS (*m*/*z*): 265 (M^+^+H). 

#### 3.2.7. ****5-Methoxycarbonyl-4-(phenyl)-6-methyl-3,4-dihydropyrimidin-2(1H)-one (4o) 

Mp 212–214°C; IR(nujol) cm^−1^: 3334 (NH), 3108 (NH), 1704 (C=O), 1650 (C=C), 1459 (CH); ^1^H NMR (300 MHz, DMSO-*d*_6_): *δ*_H_ 9.21 (br s, 1H, NH), 7.75 (br s, 1H, NH), 7.21–7.31 (m, 5H, ArH), 5.13 (s, 1H,CH), 3.51 (s, 3H, CH_3_O), 2.23 (s, 3H, CH_3_); MS (*m*/*z*): 247 (M^+^+H). 

#### 3.2.8. ****5-Ethoxycarbonyl-4-(4-methylyphenyl)-6-methyl-3,4-dihydro-pyrimidin-2(1H)-thione (4s) 

Mp 194–198°C; IR(nujol) cm^−1^: 3323 (NH), 3165 (NH), 1670 (C=O), 1575 (C=C), 1459 (CH); ^1^H NMR (300 MHz, DMSO-*d*_6_): *δ*_H_ 10.28 (br s, 1H, NH), 9.60 (br s, 1H, NH), 7.12 (d, 2H, *J* = 8.1 Hz, ArH), 7.06 (d, 2H, *J* = 8.1 Hz, ArH), 5.10 (d, 1H, *J* = 2.8 Hz, CH), 3.97 (q, 2H, *J* = 6.95 Hz, OCH_2_), 2.25 (s, 3H, CH_3_), 2.24 (s, 3H, ArCH_3_), 1.08 (t, 3H, *J* = 7.15 Hz, CH_3_); MS (*m*/*z*): 291 (M^+^+H).

#### 3.2.9. ****5-Ethoxycarbonyl-4-(4-hydroxyphenyl)-6-methyl-3,4-dihydropyrimidin-2(1H)-thione (4t) 

Mp 196–198°C; IR(nujol) cm^−1^: 3357 (NH), 3108 (NH), 1670 (C=O), 1644 (C=C), 1459 (CH); ^1^H NMR (300 MHz, DMSO-*d*_6_): *δ*_H_ 10.24(br s, 1H, OH) 9.55 (br s, 1H, NH), 9.43 (br s, 1H, NH), 6.99 (d, 2H, *J* = 8.1 Hz, ArH), 6.69 (d, 2H, *J* = 8.1 Hz, ArH), 5.04 (d, 1H, *J* = 2.8 Hz, CH), 3.98 (q, 2H, *J* = 6.9 Hz, OCH_2_), 2.26(s, 3H, CH_3_), 1.09 (t, 3H, *J* = 6.9 Hz, CH_3_); MS (*m*/*z*): 293 (M^+^+H). 

#### 3.2.10. ****5-Ethoxycarbonyl-4-(4-methoxyphenyl)-6-methyl-3,4-dihydro-pyrimidin-2(1H)-thione (4u)

Mp 140–142°C; IR(nujol) cm^−1^: 3311 (NH), 3165 (NH), 1664 (C=O), 1574 (C=C), 1459 (CH); ^1^H NMR (300 MHz, DMSO-*d*_6_): *δ*_H_ 10.28 (br s, 1H, NH), 9.59 (br s, 1H, NH), 7.12 (d, 2H, *J* = 8.6 Hz, ArH), 6.89 (d, 2H, *J* = 8.6 Hz, ArH), 5.10 (d, 1H, *J* = 3.9 Hz, CH), 3.99 (q, 2H, *J* = 7.15 Hz, OCH_2_), 3.71 (s, 3H, CH_3_O), 2.27 (s, 3H, CH_3_), 1.10 (t, 3H, *J* = 7.15 Hz, CH_3_); MS (*m*/*z*): 307 (M^+^+H).

## 4. Conclusion

In summary, we have developed an efficient, ecofriendly and solvent-free method for the synthesis of 3,4-dihydropyrimidin-2(1H)-ones **(4a–4u)**. The present method which makes use of commercially available Eaton's reagent offers a very attractive features such as shorter reaction times, simple operations with extremely milder conditions, green aspects avoiding hazardous organic solvents, toxic catalyst, and provides good to excellent yields.

## Figures and Tables

**Table 1:** Comparison of reaction conditions and yield of product **(4a)** with reported methods versus the present method.

Entry	Reagent	Condition	Time	Yield (%)	Reference
1	Methanesulfonic acid	Ethanol, reflux	1 h	95	[11]
2	P_2_O_5_	Ethanol, reflux	4 h	91	[12]
3	Chlorosulfonic acid	Solvent free, 60^°^C	30 min	93	[35]
4	P_2_O_5_/SiO_2_	Solvent free, 85^°^C	2 h	95	[36]
5	ZnCl_2_	Solvent free, 80^°^C	20 min	90	[40]
6	I_2_	Solvent free, 90^°^C	15 min	86	[41]
7	CF_3_COONH_4_	Solvent free, 80^°^C	10 min	98	[38]
**8**	**1:10 P** **2** **O** **5** **/MeSO** **3** **H**	**S** **olvent free, rt**	**5 ** **min**	**94**	—

**Table 2:** Optimization of reaction conditions for the synthesis of 3,4-dihydropyrimidin-2(1H)-ones (4k) with 4-chlorobenzaldehyde.

Entry	Reagent	Solvent	Condition	Time	Yield (%)
1	Eaton's reagent	Ethanol	RT, stir	2.3 h	60
2	Eaton's reagent	Ethanol	Reflux	2 h	65
**3**	**Eaton's reagent **	**Solvent free **	**RT,** ** stir**	**5 min**	**85**

**Table 3:** Expeditious synthesis of 3,4-dihydropyrimidin-2(1H)-ones (4a–4u) using Eaton's reagent under solvent-free conditions^a^.

Entry	R_1_	R_2_	X	Time (min)	Yield^b^ (%)	Melting point (^°^C)	
						Found	Reported [reference]
**4** **a**	C_6_H_5_	C_2_H_5_	O	5	94	202–204	202–204 [12]
**4b**	4-F–C_6_H_4_	C_2_H_5_	O	5	90	184–186	182-183 [31]
**4c**	4-NO_2_–C_6_H_4_	C_2_H_5_	O	10	89	210–212	207-208 [12]
**4d**	2,3-Cl_2_–C_6_H_3_	C_2_H_5_	O	10	91	248–250	—
**4e**	2,6-Cl_2_–C_6_H_3_	C_2_H_5_	O	10	91	284–286	280–283 [42]
**4f**	3-NO_2_–C_6_H_4_	C_2_H_5_	O	10	89	226–228	226-227 [11]
**4g**	2,4-Cl_2_–C_6_H_3_	C_2_H_5_	O	10	89	252–254	249–251 [11]
**4h**	4-Br–C_6_H_4_	C_2_H_5_	O	5	92	216–218	213–215 [43]
**4i**	4-OH–C_6_H_4_	C_2_H_5_	O	15	75	226–228	227-228 [11]
**4j**	2-Cl–C_6_H_4_	C_2_H_5_	O	5	85	218–220	216–219 [11]
**4k**	4-Cl–C_6_H_4_	C_2_H_5_	O	5	85	214–218	213–215 [12]
**4l**	4-OCH_3_–C_6_H_4_	C_2_H_5_	O	15	86	202–204	201-202 [12]
**4m**	2-Cl–C_6_H_4_	CH_3_	O	5	90	224–228	224-225 [11]
**4n**	4-F–C_6_H_4_	CH_3_	O	5	86	208–210	—
**4o**	C_6_H_5_	CH_3_	O	5	92	212–214	213-214 [11]
**4p**	4-Cl–C_6_H_4_	CH_3_	O	5	90	210–212	204–207 [11]
**4q**	C_6_H_5_	C_2_H_5_	S	5	96	208–209	208-209 [12]
**4r**	3-NO_2_–C_6_H_4_	C_2_H_5_	S	5	80	202–204	202–204 [44]
**4s**	4-CH_3_–C_6_H_4_	C_2_H_5_	S	10	77	194–198	191–193 [45]
**4t**	4-OH–C_6_H_4_	C_2_H_5_	S	15	80	196-198	195–197 [44]
**4u**	4-OCH_3_–C_6_H_4_	C_2_H_5_	S	15	77	140–142	140 [46]

^a^Aldehyde (1 mmol), ethyl/methylacetoacetate (1 mmol), urea/thiourea (1.5 mmol), Eaton's reagent (2 mmol), solvent-free, RT.

^b^Yield refers to isolated product.
